# A framework for testing pathways from prenatal stress-responsive hormones to cardiovascular disease risk

**DOI:** 10.3389/fendo.2023.1111474

**Published:** 2023-05-08

**Authors:** LillyBelle K. Deer, Chen Su, Natalie A. Thwaites, Elysia Poggi Davis, Jenalee R. Doom

**Affiliations:** ^1^Department of Psychology, University of Denver, Denver, CO, United States; ^2^Department of Psychiatry & Human Behavior, University of California, Irvine, Irvine, CA, United States

**Keywords:** cardiovascular disease (CVD), cortisol, placental CRH, health behaviors, cardiometabolic risk

## Abstract

Cardiovascular disease (CVD) is a leading cause of death globally, with the prevalence projected to keep rising. Risk factors for adult CVD emerge at least as early as the prenatal period. Alterations in stress-responsive hormones in the prenatal period are hypothesized to contribute to CVD in adulthood, but little is known about relations between prenatal stress-responsive hormones and early precursors of CVD, such as cardiometabolic risk and health behaviors. The current review presents a theoretical model of the relation between prenatal stress-responsive hormones and adult CVD through cardiometabolic risk markers (e.g., rapid catch-up growth, high BMI/adiposity, high blood pressure, and altered blood glucose, lipids, and metabolic hormones) and health behaviors (e.g., substance use, poor sleep, poor diet and eating behaviors, and low physical activity levels). Emerging evidence in human and non-human animal literatures suggest that altered stress-responsive hormones during gestation predict higher cardiometabolic risk and poorer health behaviors in offspring. This review additionally highlights limitations of the current literature (e.g., lack of racial/ethnic diversity, lack of examination of sex differences), and discusses future directions for this promising area of research.

## 1 Introduction

Cardiovascular disease (CVD) is a leading cause of death globally, killing 18.6 million people worldwide in 2019 (1). As a result, there is a strong public health imperative to identify the early factors that may predict the development of CVD or prevent its occurrence. Early risk factors for CVD, such as child obesity, atherosclerotic plaque formation, low physical activity, and poor diet can be detected early in life and contribute to poorer cardiovascular health (2–5). The Developmental Origins of Health and Disease (DOHaD) hypothesis posits that environmental exposures early in life, particularly during the prenatal period, can result in alterations in development that can have lasting health consequences for offspring (6, 7). A large epidemiological literature corroborates the DOHaD hypothesis showing that birth outcomes such as low birthweight and premature birth, are robustly associated with adult CVD (8–11). Small size at birth and premature birth do not *cause* CVD; rather, they are thought to reflect perturbations in the prenatal period that shape the development of physiological systems contributing to CVD later in life. Alterations in stress responsive hormones are one plausible mechanism by which exposures in the prenatal period affect offspring health, as stress-responsive hormones are sensitive to prenatal perturbations and also associated with poorer birth outcomes (12–20). However, less is known about relations between prenatal stress-responsive hormones and early precursors of CVD, including offspring cardiometabolic risk and health behaviors.

The current paper will review associations between prenatal stress-responsive hormones and early risk factors for later CVD, which include both cardiometabolic risk markers (e.g., rapid catch-up growth, high BMI/adiposity, high blood pressure, and altered blood glucose, lipids, and metabolic hormones) and health behaviors (e.g., substance use, poor sleep, poor diet and eating behaviors, and low physical activity levels). We begin by providing a brief overview of the dynamic changes in prenatal stress-responsive hormones focusing on the hypothalamic-pituitary-adrenal (HPA) and placental axis. We then provide a theoretical model of the relation between prenatal stress-responsive hormones and adult CVD. We present the state of the literature testing this model in both animal models, where experimental evidence is robust, and in human research. We conclude by discussing important gaps (e.g., lack of racial/ethnic diversity) and highlighting future directions for this promising area of research.

## 2 Overview of prenatal stress-responsive hormones

**Figure 1:**
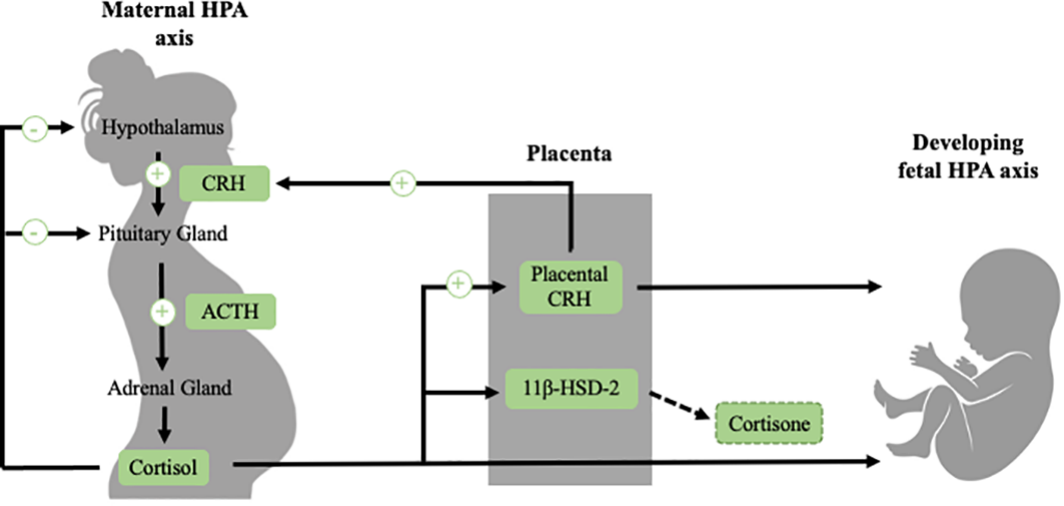
The regulation of the maternal HPA axis changes dramatically over the course of gestation, largely due to the development of the placenta, which is an organ of fetal origin. In non-pregnant individuals, exposure to a stressor activates the HPA axis, which involves the release of CRH, ACTH and cortisol. This stress system is regulated by a negative feedback loop (illustrated in black). During pregnancy (illustrated in green), CRH is released from the placenta into both the maternal and fetal compartments. In contrast to the inhibitory effect that cortisol has on hypothalamic CRH, maternal cortisol stimulates placental CRH production, producing a positive feedback loop. Placental CRH normatively increases exponentially over the course of gestation. In addition to placental CRH, maternal cortisol passes through the placenta to the fetus. However, transfer of maternal cortisol into the fetal compartment is somewhat blocked due to placental 11β-HSD-2, a placental enzyme which oxidizes cortisol into inactive cortisone. 11β-HSD-2 activity decreases late in pregnancy in order to allow maternal cortisol to reach the fetus to aid in maturation of vital organs such as the lungs. The fetal HPA axis begins to develop early in gestation and becomes increasingly active close to birth. See text for further description.

Prenatal stress-responsive hormones such as placental corticotrophin-releasing hormone (CRH) and cortisol have been posited as prenatal influences that contribute to the programming of adult CVD (21, 22). This section will provide a brief overview of the function of the HPA axis and how the HPA axis in the maternal-placental-fetal stress system changes over pregnancy (**Figure 1**). For a more comprehensive review of the maternal-placental-fetal stress system and the development of the fetal HPA axis, we refer readers to recent reviews (23, 24).

When an individual is confronted with a stressor, a cascade of physiological responses occurs to prepare the individual to cope with the stressor. When stressors are detected, neural signals are relayed to the paraventricular nucleus (PVN) in the hypothalamus, the amygdala, the hippocampus, and the locus coeruleus, which release the neuropeptides corticotropin-releasing hormone (CRH) and arginine-vasopressin (AVP) into the hypophyseal portal system (25–29). CRH and AVP stimulate the production of the prohormone proopiomelanocortin, which is cleaved by enzymes into adrenocorticotropic hormone (ACTH) and other peptides into the bloodstream (25, 30). ACTH binds to receptors in the cortices of the adrenal gland, which stimulate the production of glucocorticoid hormones from the zona fasciculata of the adrenal cortices (25, 26, 31). Cortisol, the primary glucocorticoid hormone in humans and non-human primates, binds to both mineralocorticoid receptors (MRs) and glucocorticoid receptors (GRs) throughout the body (26, 32). Corticosterone is the primary glucocorticoid in rodents and operates in a similar fashion. Under basal conditions, cortisol mainly binds to MRs due to its higher affinity, but under stress conditions it also binds to GRs. When cortisol binds to GRs, a negative feedback loop is triggered, such that in a healthy system the stress response is typically terminated (26, 32).

Over the course of gestation, many changes occur in the functioning of the maternal and the developing fetal HPA axes, largely due to the growth of a new organ, the placenta (33, 34). The placenta is a fetal organ that is responsible for changes in the maternal stress system and the development of the fetal stress system. A function of the placenta is the exchange of signals and information between the maternal and fetal stress systems. Additionally, the placenta produces a myriad of hormones into both the maternal and fetal systems, including a key stress responsive hormone CRH. Placental CRH is identical to hypothalamic CRH in its structure and bioactivity, and is an integrative stress signal that increases in response to many stressors from the maternal and fetal environment, such as nutrient restriction, infection, reduced intrauterine blood flow, and maternal depression, stress and anxiety (16, 35–38). Placental CRH normatively increases exponentially across gestation, approximately 40-fold from the end of the first trimester through term (12, 39). In contrast to the inhibitory effect that cortisol has on hypothalamic CRH, cortisol stimulates placental CRH production, producing a positive feedback loop whereby stressors in the maternal or fetal compartments that increase cortisol can stimulate the production of CRH from the placenta (40). Placental CRH plays a central role in both the regulation of fetal development and the timing of parturition (13, 33). While the normative increases in placental CRH are important for fetal maturation, accelerated production of CRH can alter fetal development. One of the most widely documented consequences of accelerated CRH production is shortened gestation and preterm birth (12, 13, 17–20, 41). Rapid increases in placental CRH may additionally alter the development of the fetal HPA axis, the brain, and have broad effects across the body (42–47).

Maternal cortisol levels also increase during pregnancy approximately three-to-five fold in comparison to pre-pregnancy levels (12, 48). Over most of gestation, transfer of maternal cortisol into the fetal compartment is partially blocked due to placental 11β-HSD-2, a placental enzyme which oxidizes cortisol into its inactive form, cortisone (49, 50). Later in gestation (around 34-35 weeks), 11β-HSD-2 activity decreases, facilitating the transfer of a greater proportion of maternal cortisol across the placenta, in order to support maturation of the fetus before birth (23, 51, 52). Placental 11β-HSD-2 additionally can be downregulated by a number of maternal stress signals such as proinflammatory cytokines, allowing a higher transfer of maternal cortisol to the fetus earlier in gestation (53, 54). Cortisol is important for fetal development, and it has been documented that cortisol levels that are too low over gestation are implicated in impaired lung (55, 56), as well as motor (57) and cognitive development (58, 59). However, levels of cortisol that are too high, especially experienced in early gestation, are also linked to altered offspring development (32, 60).

Accelerated increases in placental CRH and maternal cortisol have a role in the development of physiological systems linked to CVD. As a result, it is plausible that these prenatal hormones predict offspring cardiometabolic risk. Prenatal stress-responsive hormones are indeed implicated in fetal development and may have far-reaching influences. Prior work has documented that placental CRH may alter development of physiological systems that contribute to CVD. Placental CRH is associated with altered HPA axis activity postnatally (61, 62), as well as with physiological systems and processes involved in the development of CVD. CRH has been identified as an important regulator of adipocyte function (63), and therefore might be involved in fat storage. Additionally, placental CRH has been linked to altered brain development (44–47), which may be involved in the regulation of appetite and other health behaviors (64, 65).

Similarly, excessive exposure to glucocorticoids *in utero* has been linked to altered postnatal HPA axis activity in offspring (61, 66–68). The HPA axis is involved in metabolism and the regulation of appetite (69–71), which suggests a pathway through which glucocorticoids *in utero* may impact long-term CVD. In addition to programming of the postnatal HPA axis, exposure to glucocorticoids during gestation has been linked to multiple physiological systems and processes that are implicated in the development of CVD, including the development and accumulation of fat cells (72) and altered insulin production (73). Further, elevated glucocorticoid levels during pregnancy have been linked to altered brain and cognitive development (58, 74, 75), which may influence factors such as regulation of appetite and eating behaviors that are linked to later CVD (64, 65). The current literature provides evidence that prenatal stress-responsive hormones are involved in the development of CVD.

## 3 Current paper

**Figure 2:**
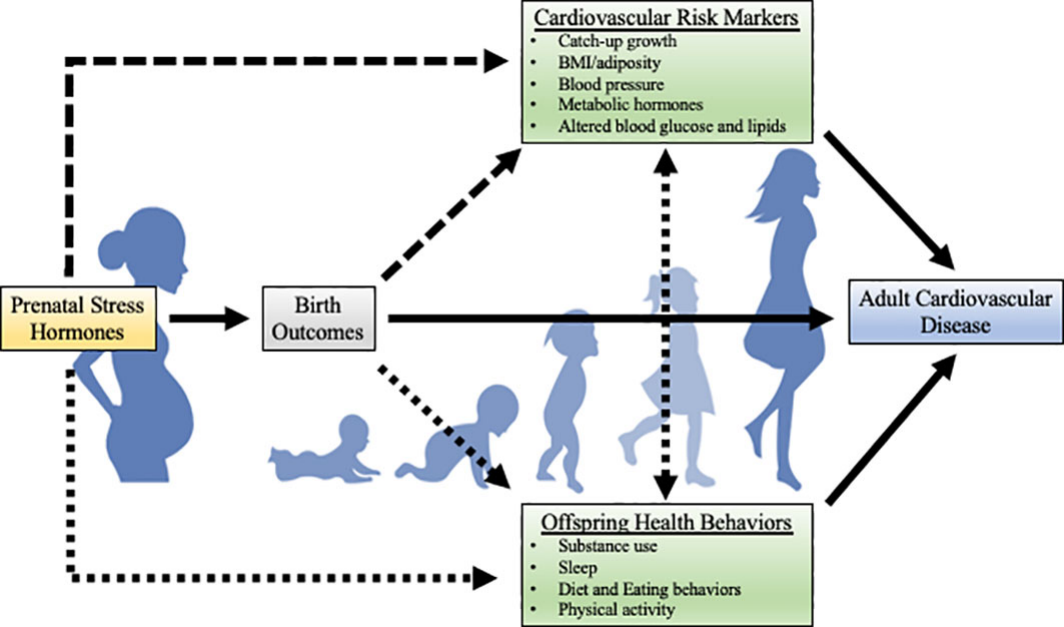
Theoretical model for potential risk factors for the development of adult CVD. The strength of the literature on each path is illustrated by the solidity of the line (solid lines indicate a larger body of research). While there are likely bidirectional relations between cardiometabolic risk markers and health behaviors, this literature is beyond the scope of the current review. Metabolic hormones include hormones such as leptin, ghrelin, and adiponectin.

In the current paper, we will discuss associations between prenatal stress-responsive hormones with risk factors for adult CVD, such as cardiometabolic risk markers (e.g., rapid catch-up growth, high BMI/adiposity, high blood pressure, and altered blood glucose, lipids, and metabolic hormones) and health behaviors (e.g., substance use, poor sleep, poor diet and eating behaviors, and low physical activity levels). **Figure 2** provides a hypothesized model for potential risk factors that may mediate the relation between prenatal stress-responsive hormones and adult CVD in offspring. The following sections will provide existing empirical evidence for each of these risk factors. In each section, research using non-human animal models, including experimental manipulation of the prenatal HPA axis, will be reviewed first. Then, observational human work will be described. Although the maternal-placental-fetal stress system is complex with many interactive factors, much of the current literature focuses on placental CRH and cortisol. As such, the current review of endogenous stress-responsive hormones will focus on placental CRH and cortisol. An important limitation to note in the human literature reviewed in this paper is that the majority of this work was conducted in largely White, WEIRD (Western, Educated, Industrialized, Rich, and Democratic) samples, limiting generalizability.

## 4 Prenatal stress-responsive hormones and offspring cardiometabolic risk markers

Researchers have begun to examine associations between prenatal stress-responsive hormones and offspring postnatal cardiometabolic risk markers (e.g., catch-up growth, high BMI and adiposity, high blood pressure, and altered glucose, lipids, and metabolic hormones such as leptin and adiponectin; 42). These cardiometabolic risk markers are robust predictors of adult CVD risk (4, 76–78). A large body of extant work tests these associations in non-human animal models. In these non-human animal model studies, administration of glucocorticoids during pregnancy is used to test causal effects of glucocorticoids on offspring outcomes. Prenatal glucocorticoid administration is linked to lower fetal growth and lower birthweight in rodent models (79–82). As described in this section, a large literature has also examined long-term effects of prenatal glucocorticoid administration in rodent animal models. A recent meta-analysis of 114 studies of prenatal glucocorticoid administration in rodents was conducted to examine the effects of prenatal maternal glucocorticoid administration on offspring cardiometabolic risk markers in adulthood, such as body mass, adiposity, systolic blood pressure, and cardiometabolic hormones (82). Although most animal work in this area has focused on glucocorticoid administration in rodents, there is parallel, yet limited work with non-human primates. There is additionally a very small literature on the link between endogenous maternal cortisol and offspring cardiometabolic risk outcomes in non-human primates. These literatures will be reviewed in the following sections.

The relation between prenatal stress-responsive hormones and offspring cardiometabolic risk has also been examined in humans (see **Table 1**). Foundational evidence establishing prenatal stress-responsive hormones as a risk factor for CVD has been conducted to examine links between hormones such as placental CRH and glucocorticoids with birth outcomes. This research documents that placental CRH is robustly implicated in fetal development and gestational timing. CRH is in the causal pathway to delivery and rapid increases in placental CRH are associated with shortened gestation and preterm birth (12, 13, 16–20), which in turn are predictors of CVD risk. Meta-analytic work also links high maternal cortisol with low birthweight (15). Similarly, research has documented a pattern of high levels of maternal endogenous cortisol early in gestation predicting higher rates of preterm birth, though this is not always consistent (14, 93–98). In parallel to this work on maternal cortisol, a few studies have examined the relation between glucocorticoid administration during pregnancy and offspring cardiometabolic risk outcomes. Glucocorticoids are administered during pregnancy if there is a high risk for preterm birth in order to accelerate fetal lung maturation (99, 100), if the pregnant person has an autoimmune or inflammatory disease (101), or for other reasons. This work demonstrates that administration of glucocorticoids prenatally are linked to lower fetal growth and birthweight (102–105). As prenatal stress-responsive hormones are linked to poor birth outcomes, and poor birth outcomes are linked to CVD, this work indicates a role for prenatal stress-responsive hormones in the development of CVD in humans as well. As described in this section, the majority of the human work links endogenous maternal cortisol levels to cardiometabolic risk markers, but there are smaller literatures that examine the effects of placental CRH and glucocorticoid administration (**Table 1**).

**Table 1:** Human studies linking prenatal stress-responsive hormones to offspring cardiometabolic risk.

Author (Year)	N	Prenatal Predictor	Predictor Gestational Timing	Offspring Outcome	Offspring Assessment Age	Finding
Stout et al. (2015) (22)	246	Endogenous placental CRH	Across gestation	BMI, catch-up growth	Infancy (0-2 years)	Exposure to higher prenatal placental CRH was associated with lower birth weight and higher rates of catch-up growth in offspring during infancy.
Hahn-Holbrook et al. (2023) (83)	189	Endogenous glucocorticoids (cortisol)	Across gestation	Rapid weight gain	Infancy (Birth – 6 months)	Trajectories of maternal cortisol over gestation characterized by high cortisol early in gestation were related to rapid increases in offspring BMI percentile over the course of infancy.
Gillman et al. (2006) (84)	199	Endogenous placental CRH	Second trimester	Adiposity	Early childhood (3 years)	Exposure to higher prenatal placental CRH was associated with smaller offspring body size but higher central adiposity in childhood.
van Dijk et al. (2011) (85)	1,320	Endogenous glucocorticoids (cortisol)	First trimester	Adiposity	Childhood (5 years)	Exposure to higher prenatal maternal cortisol was associated with higher adiposity in females and lower adiposity for males.
Laugesen et al. (2022) (86)	383,877	Glucocorticoid administration	Across gestation	BMI	Childhood (5-8 years)	Exposure to administered glucocorticoids (especially during the second trimester) predicted higher offspring BMI in males, but not females.
Hohwü et al. (2015) (87)	655	Endogenous glucocorticoids (cortisol)	Second and third trimesters	BMI	Childhood to Adolescence (2-16 years)	Exposure to higher prenatal maternal cortisol in the second trimester predicted higher offspring BMI in childhood and adolescence.
Rondó et al. (2010) (88)	130	Endogenous glucocorticoids (cortisol)	Third trimester	Arterial stiffness	Childhood (5-7 years)	Exposure to higher prenatal maternal cortisol was associated with higher offspring arterial stiffness.
Doyle et al. (2000) (89)	177	Glucocorticoid administration	Third trimester	Blood Pressure	Adolescence (14 years)	Exposure to administered glucocorticoids predicted higher offspring systolic and diastolic blood pressure.
Kelly et al. (2012) (90)	102	Glucocorticoid administration	Third trimester	Arterial stiffness	Adulthood (23-28 years)	Exposure to administered glucocorticoids predicted higher arterial stiffness.
Fasting et al. (2009) (91)	349	Endogenous placental CRH	Second trimester	Adiponectin and leptin	Early childhood (3 years)	Exposure to higher prenatal placental CRH was associated with higher offspring levels of adiponectin in childhood. There was no association with leptin.
Stinson et al. (2015) (92)	262	Endogenous glucocorticoids (cortisol)	Third trimester	Coronary heart disease risk	Adulthood (42 years)	Exposure to higher prenatal maternal cortisol was associated with higher risk of coronary heart disease in the next 10 years for female, but not in male offspring.

* Note: Participants in the noted studies were overwhelmingly from WEIRD countries (90%). Race/ethnicity was not reported in all cited studies and in the half that did report, over two thirds of the participants identify as White.

In this section, we will first describe research conducted examining the relation between prenatal stress-responsive hormones and catch-up growth (section 4.1), BMI and adiposity (section 4.2), blood pressure (section 4.3), and other cardiometabolic risk markers such as altered glucose, lipids, and metabolic hormones such as leptin and adiponectin (section 4.4). Within each section, experimental non-human animal model research will be described first, followed by observational research in humans. A summary of the literature on prenatal stress-responsive hormones and cardiometabolic risk markers in humans is summarized in **Table 1**. As the literature on this relation in animal models is quite expansive and has been covered in meta-analytic work (82), we did not include a table overviewing links between prenatal stress responsive hormones and cardiometabolic risk in animal model (see 87 for review).

### 4.1 Catch-up growth and rapid postnatal weight gain

Catch-up growth, which is a pattern of growth characterized by small size at birth followed by rapid weight gain, is a strong predictor of later obesity and cardiometabolic disease (106, 107). One study conducted with marmosets examined the relation between endogenous maternal prenatal cortisol and offspring catch-up. This study demonstrated that elevated maternal cortisol, especially early in gestation, was linked to low offspring BMI change (compared to typical BMI increases) in the early postnatal period followed by a rapid catch-up growth period that lasted into adolescence (108).

One study has examined the link between placental CRH and catch-up growth in humans. Researchers in our group investigated the relation between exposure to placental CRH at five points during pregnancy (15, 19, 25, 30, and 37 gestational weeks) with offspring BMI trajectories over the first 24 postnatal months (22). Higher placental CRH at 30 gestational weeks predicted two patterns of accelerated BMI trajectories over the first 24 months of life. The first is a *rapid-increase* BMI trajectory, which was characterized by a low BMI percentile at birth and low birthweight followed by rapid increases in BMI percentile over the first year of life. The second is a *delayed-increase* BMI trajectory, which was characterized by a low birthweight, a subsequent reduction in BMI percentile over the first year, followed by a rapid increase in BMI percentile over the second year of life. Both of these growth profiles are indicative of catch-up growth, which is linked to an increased risk of obesity and metabolic diseases (22). The association between glucocorticoids and catch-up growth has not been examined in humans, but another study examined rapid postnatal weight gain over the course of infancy, another risk factor for CVD (109). This study found that offspring exposed to trajectories of high maternal cortisol levels early in gestation that later plateau, exhibit rapid increases in BMI percentile over the course of infancy (83).

### 4.2 Body mass and adiposity

The majority of rodent animal work has examined offspring body mass and adiposity, finding an overall pattern where dams who were administered prenatal glucocorticoids had adult offspring with lower total body mass, but higher adiposity (fat mass), suggesting a profile of lower-weight animals with higher body fat (82). Similarly, two studies of male baboon offspring found that offspring who were exposed to glucocorticoid administration in mid-pregnancy had higher adiposity compared to non-exposed offspring (110, 111).

In parallel with the non-human animal model work, the majority of human research assessing the relation between prenatal stress-responsive hormones and cardiometabolic risk has examined offspring body mass and adiposity. One study conducted by Gillman and colleagues, examined the relation between placental CRH at 27 gestational weeks and both BMI and central adiposity when offspring were three years of age (84). They found that although higher placental CRH at the end of the second trimester was related to lower offspring BMI at three years of age, higher placental CRH predicted higher central adiposity, which is a risk factor for later CVD (3, 112, 113).

A few studies have tested the relation between endogenous maternal cortisol and offspring body mass and adiposity. One study found that higher maternal cortisol at the end of the first trimester (13 gestational weeks) was linked to higher offspring fat mass in females, but lower fat mass in males at 5 years of age (85). Extending out further into childhood, Hohwü and colleagues found that higher maternal cortisol at the beginning of the second trimester (16 gestational weeks) was related to a higher likelihood of offspring being overweight between 2-6 and 12-16 years, but not at 7-11 years of age, suggesting potential timing effects (87).

Lastly, in parallel to the literature examining endogenous cortisol in humans, there is one study that assessed the link between glucocorticoid administration during pregnancy and offspring overweight status between 5-8 years of age using cohort data from Denmark (86). They found that, in boys, the prenatal administration of high-dose synthetic glucocorticoids (especially during the second trimester) predicted a higher likelihood of offspring being classified as overweight or obese between 5-8 years of age compared to unexposed offspring. Taken together, both non-human animal models and research conducted in humans indicate a pattern of higher body mass and potentially higher fat mass following exposure to heightened levels of prenatal stress-responsive hormones.

### 4.3 Blood pressure

Fewer studies have examined effects of prenatal stress-responsive hormones on blood pressure in offspring. Studies reviewed in the meta-analysis of rodent model glucocorticoid administration revealed that prenatal maternal glucocorticoid administration led to higher systolic blood pressure in adult offspring (82). Research with blood pressure as an outcome in non-human primate work is less consistent. One study of adult marmosets found no effects of prenatal glucocorticoid administration in the last week of pregnancy on offspring blood pressure (114). In contrast, another study found that adult African vervet offspring who experienced glucocorticoid administration in mid-pregnancy had higher systolic and diastolic blood pressure in adulthood (115).

While the link in humans between prenatal CRH and blood pressure has not yet been examined, there are studies testing the relations between both endogenous maternal cortisol and glucocorticoid administration with offspring blood pressure. Rondó and colleagues found that higher maternal cortisol in late pregnancy predicted lower arterial elasticity when offspring were 5-7 years of age (88), indicating a higher risk for hypertension. Doyle and colleagues tested the relation between prenatal glucocorticoid administration and offspring blood pressure at 14 years of age in preterm children (89). They found that children exposed to prenatal glucocorticoid administration had both higher systolic and diastolic blood pressure at 14 years of age. One other study that examined arterial stiffness in adult offspring who had received prenatal glucocorticoids documented that this treatment is related to higher arterial stiffness (90). While this body of literature is small, it indicates that there may be a relation between prenatal stress-responsive hormones and offspring blood pressure.

### 4.4 Other cardiometabolic risk markers

Other cardiometabolic risk markers, such as metabolic hormones and altered blood glucose and lipids have also been examined. Work reviewed in the rodent animal model meta-analysis documents that glucocorticoid administration caused higher levels of the metabolic hormone leptin, which is involved in the regulation of hunger (82). There were no consistent associations between prenatal glucocorticoid administration and offspring glucose metabolism, insulin, or triglycerides in rodent animal models reviewed in that meta-analysis and no literature on these associations in non-human primates.

In humans, one study has investigated the cardiometabolic hormones adiponectin and leptin in 3-year-old children in relation to placental CRH levels (91). They found that high placental CRH in the second trimester was related to higher level of offspring adiponectin, a cardiometabolic hormone involved in insulin sensitivity and metabolism (91). There was no relation between placental CRH and leptin (91). No work to our knowledge has examined links between either endogenous maternal cortisol or glucocorticoid administration with metabolic hormones or blood glucose in humans.

One study examined an alternative way of operationalizing cardiometabolic risk in humans. In this study, the researchers examined the relation between endogenous maternal cortisol levels in the third trimester of pregnancy and risk for developing coronary heart disease in the next 10 years (Framingham risk algorithm) when offspring were 42 years of age (92). They found that, in female offspring, higher maternal cortisol levels in the third trimester predicted a higher 10-year coronary heart disease risk (92).

### 4.5 Summary – prenatal stress responsive hormones and cardiometabolic risk

The literature reviewed in this section indicates that prenatal stress-responsive hormones such as glucocorticoids and placental CRH are implicated in cardiometabolic risk (catch-up growth, high BMI and adiposity, high blood pressure, and altered blood glucose, lipids, and metabolic hormones). These signals indicate that offspring exposed to altered levels of prenatal stress-responsive hormones have higher cardiometabolic risk. The small human literature is bolstered by the robust animal model literature, where glucocorticoid levels can be manipulated through glucocorticoid administration and potential confounding variables can be tightly controlled. Further, prenatal stress-responsive hormones are strongly implicated in offspring birth outcomes, and the current literature points to a relation between offspring birth outcomes and cardiometabolic risk. As a result, this work lends further support to the hypothesis that prenatal stress-responsive hormones contribute to CVD later in life. Most of the studies described in this section examine outcomes in infancy and early childhood (22, 83–88, 91), but there are a few studies that examine adolescence (89, 116) and adulthood (90, 92). More research needs to be conducted, especially focused on endogenous maternal cortisol and placental CRH with offspring cardiometabolic risk in humans.

## 5 Prenatal hormones and offspring health behaviors

As described above, growing evidence from both research with humans and non-human animal models suggests there is an association between prenatal stress-responsive hormones and offspring cardiometabolic risk markers. However, much less is known about potential behavioral risk factors that may mediate the association between prenatal stress-responsive hormones and adult CVD. Health behaviors such as substance use, sleep, obesogenic eating behaviors, diet, and physical activity, are robust predictors of CVD (117–131). As described in the next section, there is a need for a new field of research on the role of prenatal stress-responsive hormones in the development of health behaviors relevant to CVD to fill this large gap. The following sections will describe empirical evidence for the relation between prenatal stress-responsive hormone exposure and substance use (section 5.1), sleep (section 5.2), obesogenic eating behaviors and diet (section 5.3), and physical activity (section 5.4). Within each section, experimental non-human animal model research will be described first, followed by observational research in humans. The current literature has examined both maternal endogenous cortisol and glucocorticoid administration in relation to these health behaviors but the literature on placental CRH in relation to health behaviors is sparse. A summary of the literature on prenatal stress-responsive hormones and health behaviors in non-human animal models is summarized in **Tables 2**, **3** summarizes this literature in humans.

**Table 2:** Animal model studies examining effects of prenatal stress-responsive hormones on offspring health behaviors.

Author (Year)	Species	N	Prenatal Predictor	Predictor Gestational Timing	Offspring Outcome	Offspring Assessment Age	Finding
Diaz et al. (1995) (132)	Rats	24	Glucocorticoid administration	Last week of gestation	Substance use	Childhood (3 weeks)	Offspring exposed to prenatal glucocorticoid administration exhibited increased sensitivity to amphetamines.
Bagley et al. (2019) (133)	Mice	21	Endogenous glucocorticoids (induced by stress)	Mid-gestation	Substance use	Adulthood (9 weeks)	Offspring exposed to higher endogenous glucocorticoids exhibited higher preference for cocaine.
Rodrigues et al. (2012) (134)	Rats	8	Glucocorticoid administration	Last week of gestation	Substance use	Adulthood (3-4 months)	Male offspring exposed to prenatal glucocorticoid administration exhibited an increased preference for opiates.
Hauser et al. (2007) (135)	Monkeys	12	Glucocorticoid administration	Early or late gestation	Eating behaviors	Adolescence (8-12 weeks)	Offspring exposed early in gestation to glucocorticoid administration spent more time feeding on solid foods.
Schroeder et al. (2017) (136)	Mice	10	Endogenous glucocorticoids (induced by CRH administration)	Late gestation	Eating behaviors	Adolescence (5 weeks)	Female offspring exposed to higher endogenous glucocorticoids exhibited more binge eating-like behaviors.
Diaz et al. (1995) (132)	Rats	24	Glucocorticoid administration	Last week of gestation	Physical activity	Childhood (3 weeks)	Offspring exposed to prenatal glucocorticoid administration exhibited higher rates of locomotor behavior.

**Table 3:** Human studies linking prenatal stress-responsive hormones to offspring health behaviors.

Author (Year)	N	Prenatal Predictor	Predictor Gestational Timing	Offspring Outcome	Offspring Assessment Age	Finding
Stroud et al. (2014) (137)	1,086	Endogenous glucocorticoids (cortisol)	Third trimester	Substance use	Adulthood (39 years)	Higher prenatal exposure to maternal cortisol predicted higher nicotine dependence, only for female offspring.
van Lieshout et al. (2015) (138)	84	Glucocorticoid administration	Third trimester	Substance use	Adulthood (30 years)	Low birthweight infants who were exposed to glucocorticoid administration had higher risk for substance use disorder
Räikkönen et al. (2015) (139)	54	Placental glucocorticoid mRNA expression	Across gestation	Sleep	Infancy (15.6 days)	Higher expression of placental genes that regulate fetal-placental glucocorticoid predicted poorer offspring sleep in infancy.
Chatterjee et al. (2018) (140)	594	Endogenous glucocorticoids (cortisol)	Second trimester	Sleep	Early childhood (4.8 years)	Maternal prenatal diurnal cortisol was not correlated with child sleep duration or efficiency.

*Note: Participants in the noted studies were overwhelmingly from WEIRD countries (75%). Race/ethnicity was not reported in half of the studies cited here, and in the half that did report, over 85% of the participants identify as White.

### 5.1 Substance use

There is evidence from non-human animal research that substance use might be a risk factor that mediates the relation between prenatal stress-responsive hormones and cardiovascular risk. Substance use behaviors such as cigarette smoking (118, 141, 142), excessive alcohol consumption (143, 144), and recreational drug use (117, 145), are well-documented risk factors for CVD (146). A few prior studies have examined the relation between prenatal stress-responsive hormones and substance use (132–134, 137, 138, 146). The majority of this work has been conducted in rodents, where glucocorticoids are manipulated during pregnancy (132–134). In two of these studies, pregnant rats were administered glucocorticoids during the last week of gestation. The offspring of rats that were administered glucocorticoids exhibited increased preference for amphetamines during childhood (132) and preference for opiates in adulthood (134). A third study found that adult mice who were exposed to stress-induced endogenous glucocorticoids exhibited a higher preference for cocaine (133). These findings serve as causal evidence that higher prenatal stress-responsive hormone levels increase proclivity towards substance use.

The literature testing the relation between stress-responsive hormones and substance use in humans is smaller but consistent with the experimental animal work (137, 138). In one prospective study, higher endogenous cortisol in the third trimester of pregnancy was related to higher nicotine dependence in adulthood for female offspring (137). Another study examining substance use disorder prevalence among adults with low birthweight, found that those who were exposed to prenatal glucocorticoid administration had a higher risk of substance use disorders in adulthood (138).

While the literature examining the relation between prenatal stress-responsive hormones and substance use is small, there is a larger number of studies aimed at establishing the association between prenatal exposures such as maternal stress, with offspring substance use in rodents (147–154). As prenatal maternal stress is a known activator of prenatal stress-responsive hormones such as placental CRH and cortisol (37, 155), these studies provide additional potential evidence that substance use could be a risk factor that mediates the relation between prenatal stress-responsive hormones and CVD. Studies in rodents that manipulate prenatal stress consistently find that offspring of prenatally stressed mothers exhibit increased preference to amphetamines (147), methamphetamine (149), ethanol (150), opioids (151, 152), and cocaine (153, 154). Taken together, the experimental animal and observational human research point to a relation between prenatal stress-responsive hormones and offspring substance use. As substance use is consistently predictive of CVD (117, 118, 141–146), this work positions substance use an important potential risk factor that mediates the relation between prenatal stress-responsive hormones and CVD.

### 5.2 Sleep

Poorer sleep across the lifespan is related to higher CVD risk (120–123, 156). Stress-responsive hormones are involved in the regulation of circadian rhythm (157), and HPA axis dysregulation is often implicated in sleep disorders (158), as well as normative variability in sleep (159), pointing to the importance of stress-responsive hormones in regulating sleep. However, the relation between prenatal stress-responsive hormones and offspring sleep is less known. To date, only a few studies have assessed the link between prenatal stress-responsive hormones and sleep, and both of these studies are in humans (139, 140). One study examined the relation between mRNA expression of genes that regulate fetal-placental glucocorticoid exposure (HSD2B11 and NR3C1) and infant offspring sleep quality (139). This study found that higher mRNA expression of HSD2B11 and NR3C1, indicating greater likelihood of maternal glucocorticoid transfer to the fetus, was related to poorer offspring sleep in early infancy (139). This study indicates that higher glucocorticoid transfer and production in the fetal compartment are related to poorer offspring sleep. However, another study found no link between maternal cortisol in the second trimester and both child sleep duration and efficiency in early childhood (140). Taken together, these findings suggest that more research is needed to characterize the relation between prenatal stress-responsive hormones and offspring sleep, as the current small literature is mixed.

Although the prenatal stress-responsive hormone and sleep literature is small, there is a much larger literature that aims to determine the association between prenatal exposures such as maternal stress, depression, and anxiety with offspring sleep (160–165). Two of these studies were conducted in rodents, where prenatal stress was experimentally manipulated (160, 161). In both studies, offspring of prenatally stressed mothers exhibited disruptions in typical sleep-wake patterns. Correlational human literature is consistent with this experimental non-human animal work, finding that prenatal stress (164), as well as mood disorders such as depression and anxiety (162, 163, 165, 166), predict poorer offspring sleep in infancy and childhood. Because stress and mood disorders can disrupt regulation of stress-responsive hormones during pregnancy, there may be a relation between prenatal stress-responsive hormones and poor offspring sleep. As poor sleep has been repeatedly linked to greater cardiovascular risk, this work indicates that sleep may be a risk factor that mediates the relation between prenatal stress-responsive hormones and CVD. However, more work is needed directly testing sleep as a risk factor that mediates the relation between prenatal stress-responsive hormones and CVD.

### 5.3 Offspring diet and obesogenic eating behaviors

Dietary quality and obesogenic eating behaviors are robust predictors of CVD (124–127), and there is strong evidence to suggest that stress influences diet, eating behaviors, and stress-responsive hormones (167–170). However, very little is known about the influence of prenatal stress-responsive hormones on offspring diet and eating behaviors. Currently, no research has examined the link between prenatal stress-responsive hormones and offspring dietary quality. However, two non-human animal studies provide evidence that prenatal stress-responsive hormones may influence offspring eating behaviors (135, 136). In one study, adolescent offspring of marmoset monkeys administered glucocorticoids early in gestation spent more time eating solid food in childhood (135). This increased time spent eating may indicate an increased appetite in the exposed offspring (135). In the second study, mice in late gestation were administered corticotrophin-releasing hormone in order to induce endogenous glucocorticoid production (136). Female offspring that experienced this increased prenatal glucocorticoid exposure exhibited more binge eating-like behaviors in adulthood (136). Additionally, low birthweight, which is indicative of perturbations in the prenatal period and has been linked to alterations in stress-responsive hormones (13), has been associated with increased intake of dietary fats and greater impulsive eating in early childhood (171). These studies provide preliminary evidence that prenatal stress-responsive hormones may influence offspring eating behaviors, though much more research is needed.

A related yet small human literature on other prenatal exposures related to alterations in prenatal stress-responsive hormones is consistent with this non-human animal work. Prenatal factors like maternal stress, depression, and anxiety activate prenatal stress-responsive hormones (16, 37, 38, 155, 172), which may then be associated with altered offspring diet and eating behaviors. Prenatal maternal stress has been examined in relation to offspring dietary quality and eating behaviors in humans (173, 174). Maternal stress across pregnancy is linked to lower preference for and consumption of healthy foods such as fruits, vegetables, and non-processed foods in childhood, indicating poor dietary quality (173). Similarly, maternal prenatal stress predicts greater offspring disordered eating behaviors in early adolescence (174). Given this preliminary evidence of associations between prenatal risk factors and diet/eating behaviors and robust work linking diet/eating behaviors to adult CVD, offspring diet and obesogenic eating behaviors may be promising risk factors to examine as mediators in the relation between prenatal stress-responsive hormones and adult CVD.

### 5.4 Physical activity

Low physical activity and greater sedentary behavior are well-established predictors of CVD (128–131). However, it is largely unknown whether prenatal stress-responsive hormones affect offspring physical activity levels, which could put them at risk for adult CVD. Just one study in rodents has examined physical activity levels following prenatal glucocorticoid administration (132). In this study, offspring whose mothers experienced glucocorticoid administration in the last week of gestation exhibited higher levels of locomotor behavior in childhood (132), suggesting that altered prenatal stress-responsive hormones could impact offspring motor development.

There is almost no research on the links between prenatal stress-responsive hormones and physical activity, but there are good reasons to believe that prenatal stress-responsive hormones may predict physical activity levels. High levels of stress and stress-responsive hormones are typically related to lower physical activity when they are measured concurrently (175–177). Additionally, the few studies that have examined the effects of experimentally manipulating prenatal stress in non-human animals suggest that prenatal stress is related to higher levels of offspring inactivity in both monkeys (178, 179) and rats (180). The current literature has not delved into mechanisms for this potential relation. A potential explanation is that the HPA axis is involved in the regulation of energy (181). It could be the case that individuals who have altered HPA axis activity may have less energy available for engagement in physical activity. Given this very small yet suggestive literature, it is important that future research further delve into the potential role of offspring physical activity as a potential risk factor that could mediate the association between prenatal stress-responsive hormones and offspring CVD.

### 5.5 Summary – prenatal stress-responsive hormones and health behaviors

The body of literature covered in this section illustrates that we know very little about the role of prenatal stress-responsive hormones in the development of each of these health behaviors (substance use, sleep, diet and obesogenic eating behaviors, and physical activity). What we do know indicates that offspring exposed to altered levels of prenatal stress-responsive hormones may be more likely to engage in substance use, poorer sleep, poorer diet, obesogenic eating behaviors, and lower physical activity. However, the very small number of studies in this area indicates a huge gap in the literature and opportunity for exciting further work to elucidate the developmental pathways to CVD. Due to suggestive research on prenatal hormones and other prenatal risk factors (stress, depression, anxiety) predicting each of these health behaviors, we argue that this area of research presents many opportunities to test the role of these behavioral risk factors in the development of adult CVD. Further, it is likely that these health behaviors are related. For example, there is robust evidence of a bidirectional association between sleep and physical activity (182). Therefore, future research should also examine potential interactions and temporal associations between these health behaviors.

## 6 Discussion

As stated throughout this review, much more research is needed to fully understand the role of prenatal stress-responsive hormones in the development of CVD. While there is a more robust animal literature, there are a small number of studies on the relation between prenatal stress-responsive hormones and cardiometabolic risk in humans. The behavioral pathways to CVD have been overlooked, as there are even fewer studies examining health behaviors as potential mediators between prenatal stress-responsive hormones and offspring cardiometabolic risk. This area presents a rich potential for future work to fully characterize these possible developmental mechanisms.

There are many unanswered questions regarding the role of prenatal stress-responsive hormones in the development of CVD. We highlight here several needed directions for future research. Studies that examine trajectories of stress-responsive hormones across pregnancy rather than single timepoints are needed. Recent work documents that there are distinct trajectories of stress-responsive hormones and that these trajectories better predict offspring outcomes than single timepoint measures (83, 155). Additionally, most of the studies reviewed here assess outcomes at just one timepoint. A developmental approach is needed, as the current literature, in which outcomes are assessed at one timepoint, precludes identification of when alterations in risk markers and health behaviors begin. A related aspect of needed research are studies that consider the sensitive period of adolescence. In the non-human animal literature reviewed here, most studies examined outcomes when the offspring were adults. In the human literature, most studies evaluated offspring either early in childhood or in adulthood, with notable gaps in late childhood and adolescence. Recent work documents that adolescence may be a window of plasticity, as the HPA axis goes through a potential recalibration through puberty to match current environmental conditions following early stress (183, 184). Examining the pubertal period in relation to prenatal stress-responsive hormone regulation may be an important period for understanding the emergence of cardiometabolic risk and health behaviors. Future developmentally-informed work will provide opportunities to create interventions to improve health at multiple developmental periods.

There is a critical gap in our understanding of sex differences in the impact of prenatal experiences on development of CVD. There are known, dramatic sex differences in CVD risk and CVD-related mortality, such that while males and females develop CVD at the same rate, males are more likely to develop and die from CVD earlier in life (185, 186). Additionally, the presentation of CVD differs between males and females (185, 187–189). Importantly, there are also well-documented sex differences in fetal responses to adversity and stress-responsive hormones, as well as in fetal growth (190–195). Male fetuses are larger than females, and prior work documents that this is partially due to male fetuses prioritizing the energy demands of growth (192, 193). It is thought that this may make male fetuses less adaptable to prenatal adversity, potentially putting them at higher risk for adverse outcomes (190–195). Given these documented differences, it is surprising that very few studies in the literature reviewed here examine sex differences. Additionally, many studies with non-human animals reviewed here only included males. This lack of examination precludes a full understanding of the developmental pathways to CVD and potential targets for prevention efforts. In addition to the need to examine sex differences, the field would benefit from a standardization of covariates used in order to facilitate replicability and consistent findings. Covariates that are commonly used in the literature reviewed here and that we recommend utilizing include gestational age at the time of the prenatal stress-responsive hormone collection, child sex, and maternal factors (e.g., BMI, socioeconomic status, age, substance use during pregnancy).

Additionally, it is imperative that future work in humans is conducted in diverse samples. The overwhelming majority of the current human literature reviewed in this paper was conducted in WEIRD countries. When studies did report race/ethnicity, the participants were overwhelmingly White. Only a few of the studies reviewed included racially and ethnically diverse populations (22, 83, 84, 91). As there are known racial disparities in CVD risk (196, 197), it is imperative for research to include minoritized populations.

The literature could also benefit from a cross-species approach. Cross-species research allows for the parallel examination of experimental animal work and observational human work. This approach allows for the disentanglement of many factors that cannot be controlled in human research, such as shared genes, other prenatal influences than stress-responsive hormones, and postnatal influences such as parenting. This approach has been impactful in other areas (198–200) and would be useful here.

While the focus of the current paper was to review the potential influences of prenatal stress-responsive hormones in the development of offspring CVD, we recognize that these hormones do not exert their influence in isolation. It is likely that prenatal exposures such as environmental toxin exposure (201–203), maternal physical health (204–206), and maternal inflammation (207–209) impact the development of CVD. There is a need for future research examining the interaction of these factors. Further, environmental context, including stress, socioeconomic status, racism and discrimination, likely influence these stress-responsive hormones (16, 155, 210, 211). Finally, the continuity/discontinuity between the prenatal and postnatal environment may profoundly impact health (193, 212–214). Thus, there is a need for research evaluating the joint role of the pre- and postnatal environments to predict CVD.

## 7 Conclusions

Converging research across non-human animal and human research indicates that prenatal stress-responsive hormones have a role in the development of offspring CVD, potentially through intermediary cardiovascular risk and altered health behaviors. Altered levels of prenatal stress-responsive hormones are implicated in higher offspring cardiometabolic risk, and potentially, CVD-related health behaviors such as substance use, poor sleep, poor diet and eating behaviors, and physical activity. Continued research examining trajectories of prenatal hormones and offspring outcomes in diverse samples is an exciting opportunity for future research.

## Author contributions

LKD, EPD, and JRD conceptualized the review. LKD, CS, NAT, EPD, and JRD drafted the manuscript. LKD and NAT drafted the figures and LKD and CS drafted the tables. All authors contributed to the article and approved the submitted version.
